# A genetic polymorphism in *P2RY*_*1*_ impacts response to clopidogrel in cats with hypertrophic cardiomyopathy

**DOI:** 10.1038/s41598-021-91372-3

**Published:** 2021-06-15

**Authors:** Yu Ueda, Ronald H. L. Li, Nghi Nguyen, Eric S. Ontiveros, Samantha L. Kovacs, Maureen S. Oldach, Karen M. Vernau, Michael H. Court, Joshua A. Stern

**Affiliations:** 1 Department of Clinical Sciences, College of Veterinary Medicine, North Carolina State University, Raleigh, NC USA; 2 Department of Veterinary Surgical and Radiological Sciences, School of Veterinary Medicine, University of California – Davis, Davis, CA USA; 3 Department of Medicine and Epidemiology, School of Veterinary Medicine, University of California – Davis, Davis, CA USA; 4 Department of Veterinary Clinical Sciences, College of Veterinary Medicine, Washington State University, Pullman, WA USA

## Abstract

Clopidogrel is converted to its active metabolite by cytochrome P450 isoenzymes and irreversibly inhibits platelet activation by antagonizing the adenosine-diphosphate (ADP) receptor. It is frequently used in cats with hypertrophic cardiomyopathy (HCM) to prevent thromboembolic complications. However, significant interpatient variability of the response to clopidogrel therapy has been suspected. In this study, we assessed the impact of single nucleotide polymorphisms (SNPs) within ADP receptor (*P2RY1, P2RY12*) and cytochrome P450 isoenzyme (*CYP2C41*) genes on platelet inhibition by clopidogrel administration in cats with HCM. Forty-nine cats completed the study, and blood samples were obtained before and after clopidogrel therapy to assess the degree of platelet inhibition based on flow cytometry and whole blood platelet aggregometry. Plasma concentrations of clopidogrel metabolites were measured after the last dose of clopidogrel. Whole blood platelet aggregometry revealed a significant reduction of platelet inhibition by clopidogrel in cats with the P2RY1:A236G and the P2RY12:V34I variants. The association with the P2RY1:A236G variant and clopidogrel resistance remained significant after adjustment for multiple comparisons. This study demonstrated that a genetic polymorphism in the *P2RY1* gene altered response to clopidogrel therapy and suggests that clinicians may consider alternative or additional thromboprophylactic therapy in cats with the P2RY1:A236G variant.

## Introduction

Arterial thromboembolism (ATE) is a common complication with high morbidity and mortality in cats with cardiomyopathy. Previous studies showed that 6–17% of cats with hypertrophic cardiomyopathy (HCM) or other cardiac diseases develop ATE with an associated mortality rate of 61–67%^1–6^. Clopidogrel is a commonly prescribed antiplatelet medication used in people as well as in veterinary patients. Clopidogrel, once metabolized to active metabolite by cytochrome P450 enzymes, irreversibly inhibits one of the platelet adenosine diphosphate (ADP) receptors, P2Y_12_. Several large human clinical trials showed that clopidogrel significantly reduces the chance of developing thromboembolic diseases^7–9^. In cats, one recent randomized controlled trial reported clopidogrel administration in cats with cardiogenic ATE significantly reduced the chance of developing recurrent thromboembolic events compared to cats receiving aspirin^10^. However, despite broad usage of clopidogrel in both human and veterinary patients, significant interpatient variability of pharmacodynamic response has been reported. In humans, clopidogrel resistance, defined by decreased inhibition of ADP-induced platelet aggregation in response to clopidogrel, was reported in 5–30 percent of people^11–13^. The aforementioned clinical trial in cats also showed frequent recurrence of ATE despite clopidogrel therapy and underscores the potential for variable response to this drug in clinical patients^10^. In addition to these findings, recent studies also reported interindividual variability of platelet inhibition by clopidogrel assessed by platelet aggregometry and flow cytometry in cats with and without hypertrophic cardiomyopathy (HCM)^14,15^.

There are various intrinsic and extrinsic causes of clopidogrel resistance. Genetic polymorphisms in both platelet ADP receptor genes (*P2RY1, P2RY12*) and the cytochrome P450 enzyme gene have been reported as inciting causes of clopidogrel resistance in people^16–24^. In cats, multiple non-synonymous single nucleotide polymorphisms (SNPs) in the *P2RY1* (NCBI ID: 101090795) and *P2RY12* genes (NCBI ID: 101094044) were identified via whole genome sequencing and Sanger sequencing verification, and two non-synonymous SNPs, P2RY1:A236G and P2RY12:V34I, were found with relatively high minor allele frequencies in a population of cats with HCM^25^. The cytochrome P450 2C (*CYP2C*) genes, were reported^26,27^. One recent study also reported that one of the variants in the feline *CYP2C41* gene (*CYP2C41* (NCBI ID: 101089396) was associated with increased clopidogrel active metabolite (CAM) concentration after administration of a single dose of clopidogrel in healthy cats^28^. This finding supports the contention that these SNPs could alter the response to clopidogrel in cats. It is, however, unknown if these variants in the *P2RY1*, *P2RY12*, and *CYP2C41* genes were associated with significant interindividual variability in cats with HCM, especially after long-term clopidogrel therapy. A better understanding of the impact of genetic polymorphisms on the inhibitory effects of clopidogrel could open doors for precision medicine and optimization of antithrombotic strategies in cats at risk of ATE.

In this study, we hypothesized that genetic polymorphisms in the genes encoding the feline platelet ADP receptors, P2RY1:A236G and P2RY12:V34I and the feline cytochrome P450 2C enzyme, CYP2C41:H231R, are common and responsible for clopidogrel resistance in cats with HCM. To test this hypothesis, the frequencies of the non-synonymous SNPs in client-owned cats with HCM were determined. Next, pharmacodynamic response to clopidogrel was evaluated by flow cytometry and whole blood platelet aggregometry before and after a 10–14 day course of clopidogrel therapy. Plasma concentrations of clopidogrel and clopidogrel metabolites including CAM were measured at the completion of the study period to determine if clopidogrel resistance were associated with altered metabolism of clopidogrel by cytochrome P450 enzymes. The results were then compared in HCM cats with and without the nonsynonymous SNPs identified in the targeted genes.

## Results

### Signalment and patient demographics

A total of 51 client-owned cats with HCM were enrolled and 49 cats completed the study. Of the 49 cats, 38 were Domestic Short Hair, 7 were Domestic Long Hair, 2 were Sphynx, 1 was Maine Coon, and 1 was Siamese. One cat was excluded from the study due to poor animal compliance resulting in the failure to administer clopidogrel at home. Another cat was excluded as there was no concentration of clopidogrel detected in plasma after completion of the study, suggesting either poor animal compliance or a failure to follow the drug administration protocol. The mean and standard deviation (SD) of age was 6.5 (± 4.5) years old with 35 castrated males and 14 spayed females. The mean body weight (± SD) was 5.6 (± 1.4) kg. No adverse effects of clopidogrel administration were reported by the owners throughout the study period.

### Missense SNPs in P2RY1, P2RY12, and CYP2C41 genes

In the present study, the frequencies of the missense SNPs in the *P2RY1, P2RY12*, and *CYP2C41* genes were successfully determined in all cats and the results are listed in Table 1. The obtained genotype distributions did not deviate from Hardy–Weinberg equilibrium (Table 1).

**Table 1:** Frequencies of genetic polymorphisms in three targeted genes (*P2RY1*, *P2RY12*, and *CYP2C41*).

Genes	Location	Mutations	Polymorphisms	Distributions n/49 (%)	HWE (Chi^2^)
*P2RY1*	C2.110,685,285	p.A236G	c/c	16/49 (32.7)	0.11
			c/g	25/49 (51.0)	
			g/g	8/49 (16.3)	
*P2RY12*	C2.112,204,070	p.V34I	g/g	23/49 (46.9)	0.16
			g/a	22/49 (44.9)	
			a/a	4/49 (8.2)	
*CYP2C41*	D2.54,712,168	p.H231R	g/g	20/49 (40.9)	0.54
			g/a	23/49 (46.9)	
			a/a	4/49 (8.2)	

HWE; Hardy–Weinberg equilibrium.

### Adenosine diphosphate-induced platelet aggregation is inhibited by clopidogrel therapy

**Figure 1:**
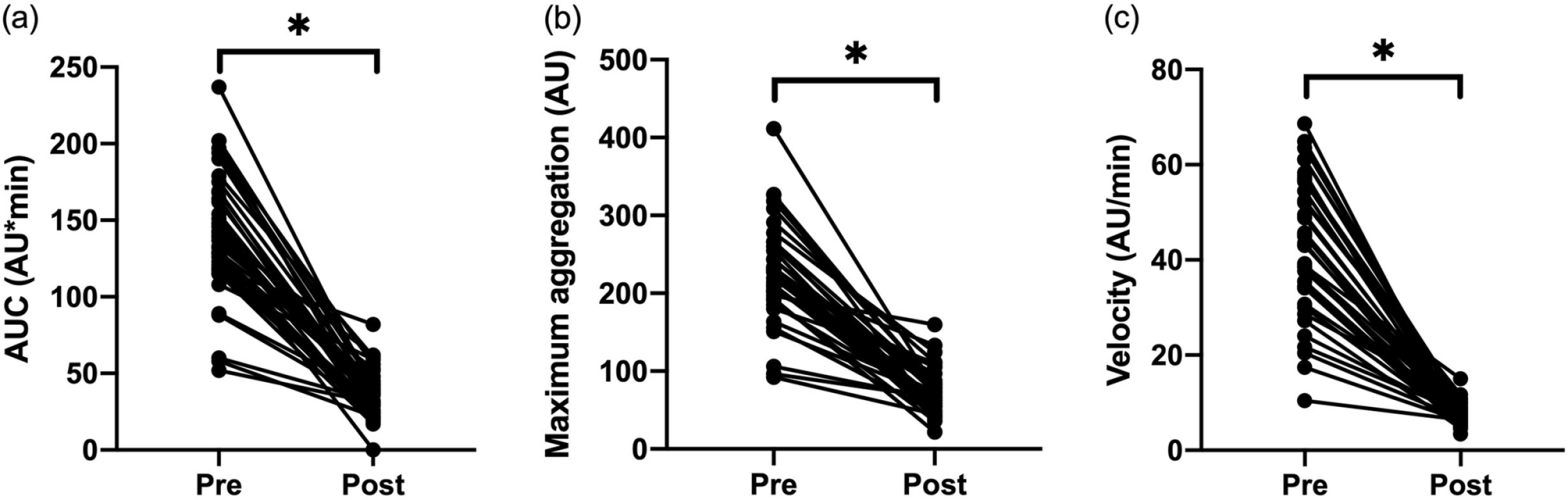
Clopidogrel therapy modulates ADP-induced platelet aggregation in cats with HCM. Platelet aggregation in response to 20 µM ADP measured before (pre) and after (post) clopidogrel therapy is shown as (**a**) area under the curve (AUC), (**b**) maximum aggregation, and (**c**) aggregation velocity. *P < 0.0001.

The mean area under the curve (AUC), maximum aggregation (AU), and aggregation velocity (AU/min) before clopidogrel therapy were 140 ± 40.2 (AU*min), 225.3 ± 63.3 (AU), and 42.9 ± 14.2 (AU/min), respectively. After clopidogrel therapy, mean AUC, aggregation, and velocity were significantly reduced at 35.9 ± 15.2 (AU*min), 73.3 ± 28.9 (AU), 7.4 ± 2.3 (AU/min), respectively (Fig. 1). The percent inhibition of AUC, maximum aggregation, and aggregation velocity were 73.6 (± 11.3) %, 66.6 (± 12.0) %, and 81.5 (± 7.8) %, respectively. The Multiplate variables were all significantly decreased after 10–14 days of clopidogrel therapy compared to pre-treatment values (*P* < 0.0001) (Fig. 1).

### Clopidogrel inhibits platelet P2Y_12_ by activating cyclic adenosine monophosphate and subsequent phosphorylation of vasodilator-stimulated phosphoprotein in cats with HCM

**Figure 2:**
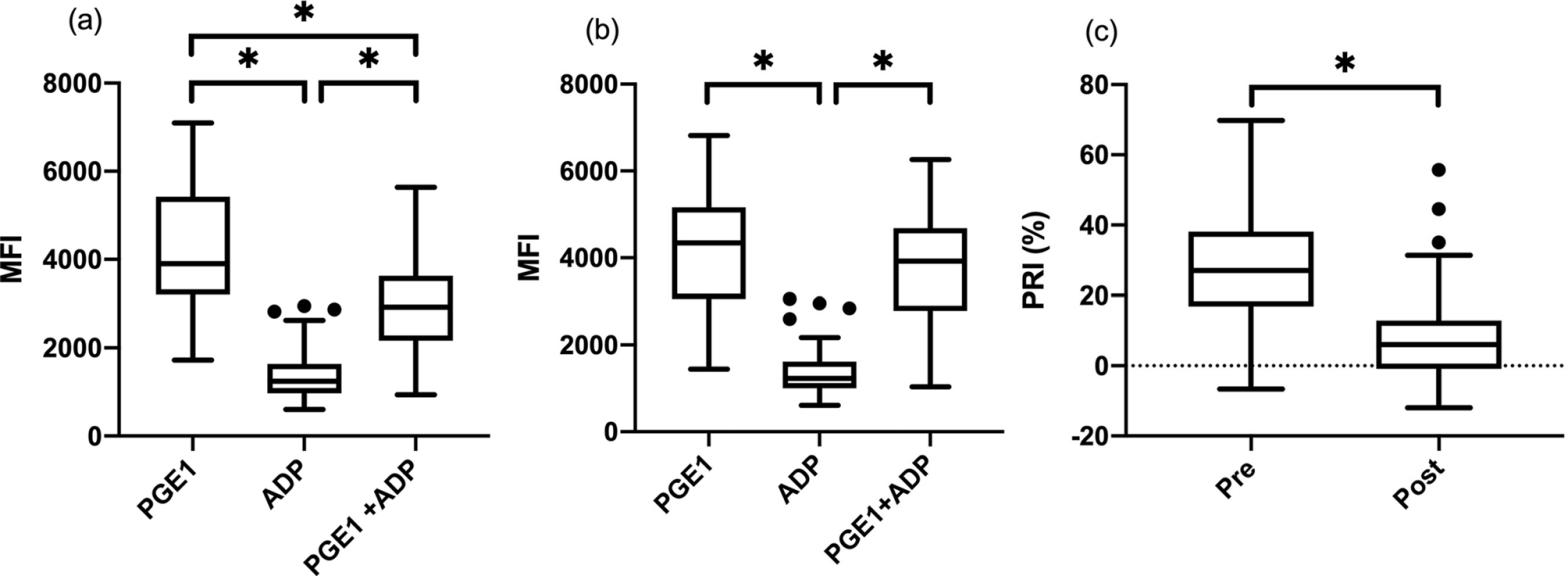
Flow cytometric analysis of intraplatelet P-VASP (**a**) before clopidogrel therapy and (**b**) after clopidogrel therapy in the presence of PGE_1_, ADP stimulation, or a combination of PGE_1_ and ADP. Note there was a significant difference between the mean fluorescence intensity (MFI) with PGE_1_ and PGE_1_ in the presence of ADP stimulation before clopidogrel therapy, but no significant difference was found after clopidogrel therapy. (**c**) Platelet reactivity index (PRI) was calculated and compared before (pre) and after (post) clopidogrel therapy. The horizontal line represents the median, the box the 25th and 75th percentile, the whiskers the 1.5 times interquartile range, and the points outside the whiskers are outliers. *P < 0.0001.

Irreversible inhibition of platelet ADP receptor, P2Y_12,_ was assessed by measuring the degree of phosphorylation of vasodilator-stimulated phosphoprotein (P-VASP) within platelets in the presence of prostaglandin E1 (PGE_1_), adenosine diphosphate (ADP) or both. PGE_1_ treated platelets served as positive control. P-VASP expression was reported with mean fluorescence intensity (MFI) measured by flow cytometry. Prior to starting clopidogrel administration, significantly lower levels of P-VASP expression was detected in ADP-activated platelets (MFI 1238, interquartile rage [IQR] 963.8–1632) compared to PGE_1_-treated platelets (MFI 3906, IQR 3201–5422) (*P* < 0.0001) (Fig. 2a). P-VASP expression was also significantly lower in PGE_1_ and ADP-treated platelets (MFI 2924, IQR 2165–3631) than PGE_1_-treated platelets (*P* < 0.0001) but higher than ADP-treated platelets (*P* < 0.0001) (Fig. 2a). These results indicate that activation of the P2Y_12_ receptor by ADP inhibits the adenyl cyclase-cyclic AMP pathway leading to lower P-VASP in platelets. After clopidogrel therapy, the P-VASP in platelets activated by ADP in the presence of PGE_1_(MFI 3925, IQR 2785–4680) was similar to that in PGE1-treated platelets (MFI 4344, IQR 3054–5164) (*P* = 0.16) (Fig. 2b) indicating adequate inhibition of P2Y_12_ receptor. Platelets activated by ADP maintained the significantly lower level of P-VASP (MFI 1229, IQR 2785–4680) than platelets activated by PGE_1_ and both PGE_1_ and ADP (*P* < 0.0001) (Fig. 2b). The mean platelet reactivity index (PRI) calculated from P-VASP expression level was compared before and after clopidogrel therapy. The median (IQR) PRI of P-VASP before and after clopidogrel therapy were 27.0% (16.9–38.2) and 6.1% (− 0.85–12.9), respectively. The PRI was significantly lower in platelets measured after clopidogrel therapy than those measured before the treatment (*P* < 0.0001) (Fig. 2c).

### Clopidogrel inhibits ADP-mediated alpha-granule secretion in cats with HCM

**Figure 3:**
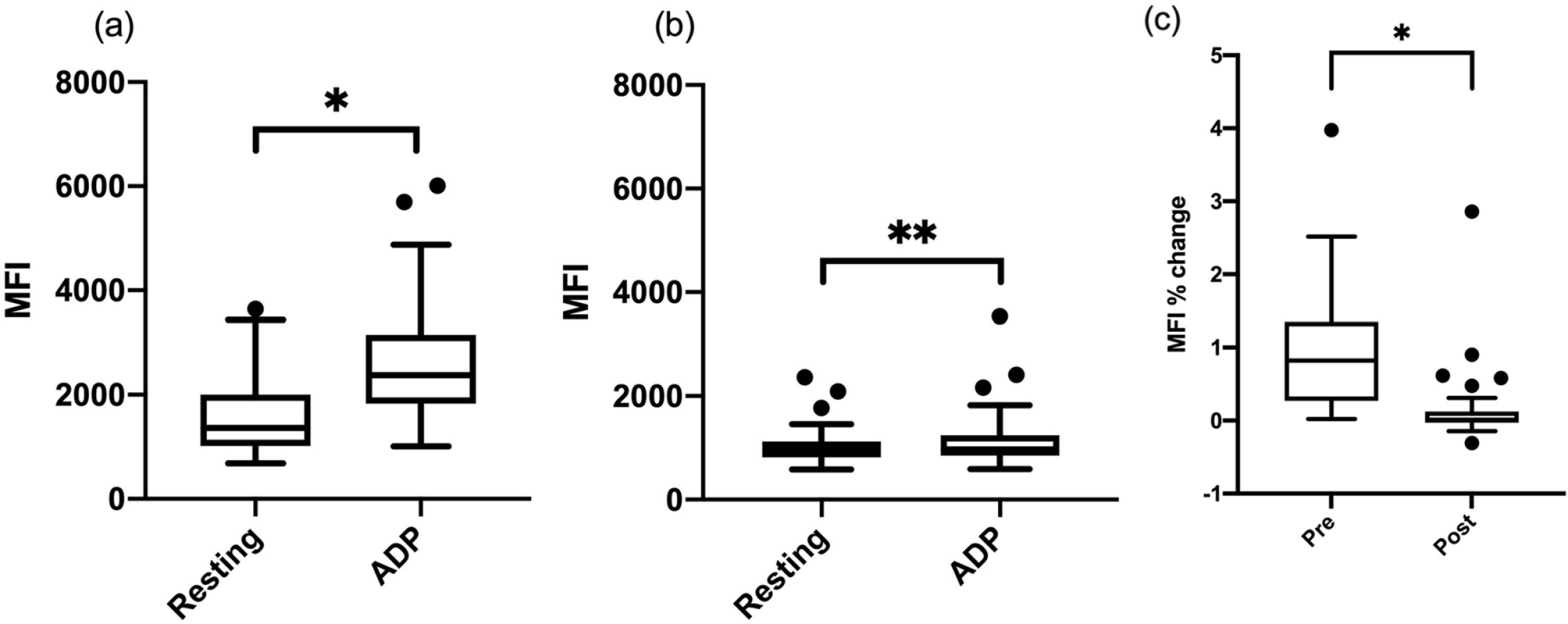
Clopidogrel inhibits platelet alpha-granule secretion in cats with HCM. Flow cytometric analysis of platelet P-selectin expression (reported with mean fluorescence intensity [MFI]) in unstimulated (resting) and ADP-stimulated status in 49 cats (**a**) before clopidogrel therapy and (**b**) after clopidogrel therapy. (**c**) Platelet reactivity to ADP calculated based on the percent (%) change in response to ADP was compared between pre-clopidogrel and post-clopidogrel therapy (*P* < 0.0001). The horizontal line represents the median, the box the 25th and 75th percentile, the whiskers the 1.5 times interquartile range, and the points outside the whiskers are outliers. *P < 0.0001, **P = 0.0084.

Platelet surface P-selectin expression was measured by flow cytometry to determine if clopidogrel modulates alpha granule secretion. The percentage of platelets positive with P-selectin expression and the MFI of P-selectin expression were compared between resting (unstimulated) and ADP-stimulated platelets. Prior to clopidogrel therapy, the median percentage of platelets with P-selectin expression were 42.1% (IQR 25.3–61.2) in resting platelets and 61.7% (IQR 44.6–72.0) in ADP-treated platelets. The median MFI of P-selectin expression was 1364 (IQR 1017–2003) in resting platelets and 2378 (IQR 1830–3151) in ADP-stimulated platelets. The ADP stimulation resulted in a significantly higher percentage of P-selectin positive platelets (*P* < 0.0001) and P-selectin MFI (*P* < 0.0001) (Fig. 3a).

After clopidogrel therapy, the median percentage of resting platelets with P-selectin expression [30.7% (IQR 15–48.6)] was not significantly different from that of ADP-activated platelets [30% (IQR 14.6–46.1)] (*P* = 0.4). However, P-selectin MFI in ADP-activated platelets [967 (IQR 853–1242)] were significantly higher than in resting platelets [960.5 (IQR 814–1126)] (*P* = 0.0084) (Fig. 3b). To determine platelet reactivity to ADP, the percent change in P-selectin MFI with or without ADP stimulation was calculated by dividing the difference between MFI with ADP stimulation and resting MFI (MFI without stimulation) by the resting MFI. The MFI percent change was then compared before and after clopidogrel therapy, and it was found to be significantly lower after clopidogrel therapy than before clopidogrel therapy (*P* < 0.0001) (Fig. 3c).

### Clopidogrel and clopidogrel metabolite concentrations do not correlate with clopidogrel-mediated platelet inhibition

Concentrations of clopidogrel, clopidogrel carboxylic acid, and derivatized clopidogrel active metabolite (CAM-D) were measured in plasma obtained from cats approximately 2 h after the last dose of clopidogrel. CAM-D metabolic ratio was calculated based on the molarity of clopidogrel, clopidogrel carboxylic acid, and CAM to minimize the impact of variable absorption of parent drugs^28^. The mean (± SD) concentrations of clopidogrel, clopidogrel carboxylic, and CAM-D were 33.8 ± 33.9 ng/mL, 612.8 ± 1303.3 ng/mL, and 15.2 ± 13.9 ng/mL, respectively. The mean (± SD) of CAM-D metabolic ratio were 0.8 (± 0.96) %. The correlation analyses between the concentrations of clopidogrel and clopidogrel metabolites and platelet function were performed, and weakly or moderately significant correlations were observed between CAM-D metabolic ratio and platelet function assessed by whole blood impedance aggregometry and P-selectin expression (Supplement Table 1).

### Genetic polymorphisms alter inhibition of platelet aggregation by clopidogrel in cats with HCM

**Figure 4:**
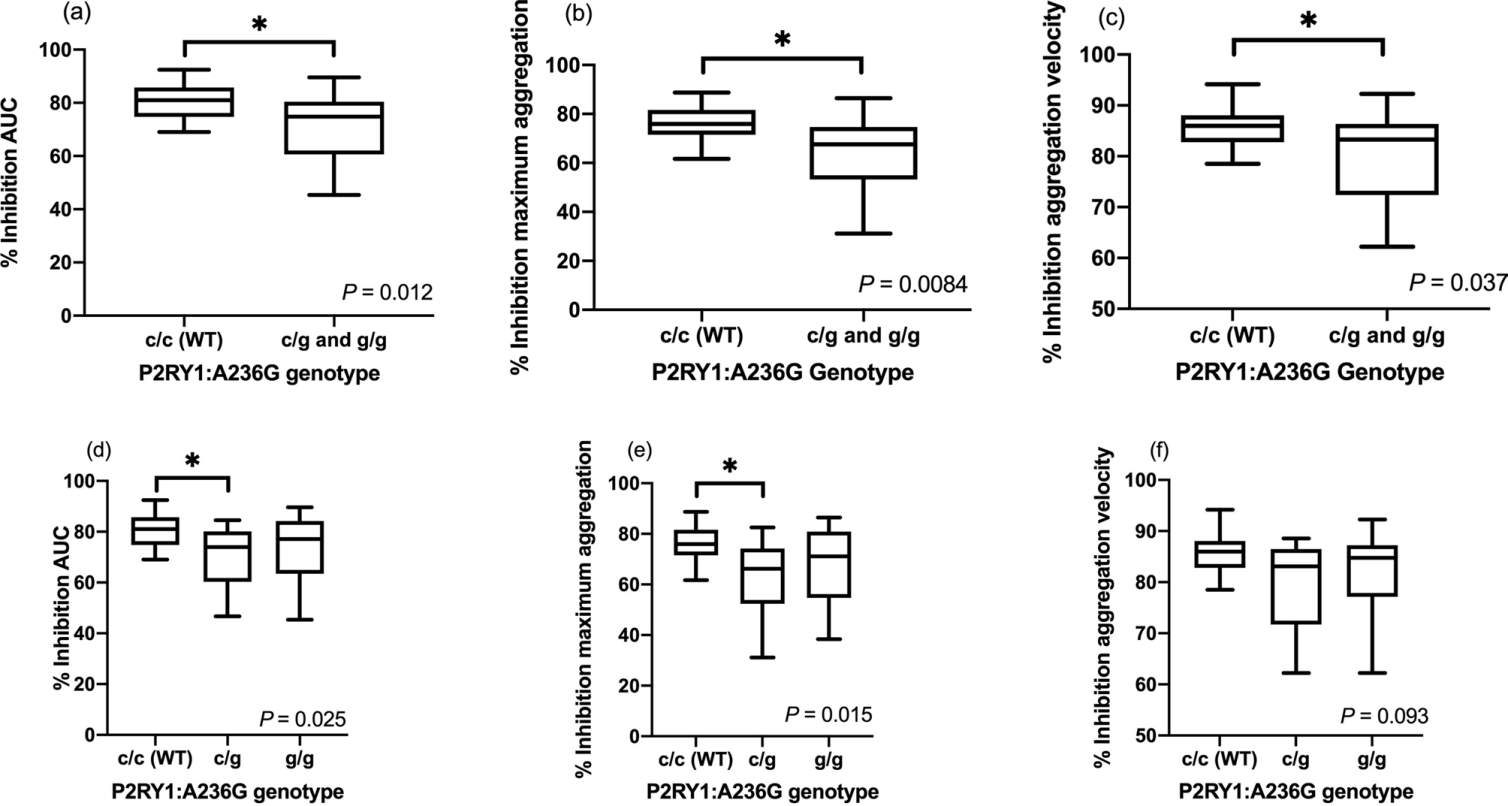
The percent inhibition of (**a**) area under the curve (AUC), (**b**) maximum aggregation (AU), and (**c**) aggregation velocity, measured by whole blood aggregometry, in cats with P2RY1:A236G wildtype (WT) (c/c) and variant genotype (both c/g and g/g genotypes). The percent inhibition of (**d**) AUC, (**e**) AU, and (**f**) aggregation velocity were further divided into wildtype (c/c), heterozygous (c/g), and homozygous (g/g) genotypes. The horizontal line represents the median, the box the 25th and 75th percentile, the whiskers the 1.5 times interquartile range, and the points outside the whiskers are outliers.

Statistical analyses were performed to determine the associations between genetic polymorphisms in *P2RY1*, *P2RY12*, *CYP2C41* genes, and clopidogrel-mediated platelet inhibition. Platelet inhibition by clopidogrel was measured as percent inhibition of AUC, maximum aggregation, and aggregation velocity based on the results of whole blood platelet aggregometry. The percent inhibitions of AUC (*P* = 0.012), maximum aggregation (*P* = 0.0084), and aggregation velocity (*P* = 0.037) were all significantly lower in cats with P2RY1:A236G variants (combined homozygous and heterozygous variants) compared to cats with P2RY1:A236G wildtype. When the percent inhibition of AUC was compared among P2RY1:A236G wildtype and heterozygous and homozygous genotypes, the percent inhibition was significantly different overall (*P* = 0.034), and the percent inhibition between the wildtype and heterozygous remained significantly different as well (*P* = 0.025). However, no significant difference of the percent inhibition was noted between the wildtype and homozygous genotypes (*P* = 0.85) and between heterozygous and homozygous genotypes (*P* > 0.99) (Table 2, Fig. 4a,d). The same comparisons were made for percent inhibition of maximum aggregation and aggregation velocity. When the percent inhibition of aggregation was compared among P2RY1:A236G wildtype and heterozygous and homozygous genotypes, they remained significantly different overall (*P* = 0.019), and the percent inhibition between the wildtype and heterozygous remained significantly different (*P* = 0.015). However, no significant difference was noted between the percent inhibition of wildtype and homozygous genotypes (*P* = 0.74) as well as between heterozygous and homozygous genotypes (*P* > 0.99) (Table 2, Fig. 4b,e). When the percent inhibition of aggregation velocity was compared among P2RY1:A236G wildtype and heterozygous and homozygous genotypes, they were not significantly different (*P* = 0.093) (Table 2, Fig. 4c,f).

**Table 2:** The percent inhibition (interquartile range [IQR]) of area under the curve (AU*min), maximum aggregation (AU), and aggregation velocity (AUC/min), measured by whole blood aggregometry, in cats with the wildtype (WT) and the variant genotype (both homozygous and heterozygous) in various genotypes (P2RY1:A236G, P2RY12:V34I, and CYP2C41:H231R).

Genotype	Nucleotide	AUC (AU*min) (IQR)	*P* value (overall)	*P* value	Maximum aggregation(AU) (IQR)	*P* value (overall)	*P* value	Aggregation velocity (AU/min) (IQR)	*P* value (overall)
P2RY1:A236G	c/c	81 (74.8–85.7)	0.03	c/c vs c/g: 0.025	76 (71.6–81.6)	0.019	c/c vs c/g: 0.015	86 (82.8–88.1)	0.093
	c/g	77.7 (71.4–80.8)		c/c vs g/g: 0.85	66.2 (52.3–74.2)		c/c vs g/g: 0.74	83.1 (71.7–86.5)	
	g/g	85.2 (82.5–89.6)		c/g vs g/g: > 0.99	71.1 (54.7–80.8)		c/g vs g/g: > 0.99	84.8 (77.1–87.3)	
P2RY12:V34I	g/g	72.9 (63.1–80.3)	0.034	g/g vs g/a: 0.96	66 (53.2–74.1)	0.019	g/g vs g/a: 0.36	82.5 (75.4–87.4)	0.35
	g/a	77.7 (71.4–80.8)		g/g vs a/a: 0.031	72 (64.8–76.3)		g/g vs a/a: 0.024	83.8 (78.5–86.4)	
	a/a	85.2 (82.5–89.6)		g/a vs a/a: 0.13	81 (79–86.5)		g/a vs a/a: 0.21	85.6 (85.6–92.3)	
CYP2C41:H231R	g/g	74.8 (69.5-78.3)	0.28		68.3 (60.2 - 71.6)	0.49		84.6 (82.1 - 85.6)	0.38
	a/g	73.8 (61.7–80.9)			70 (55–75.8)			81.2 (71.8–86.5)	
	a/a	80.3 (65.1 - 83.0)			74.2 (60.1 - 80.4)			84.4 (82.4 - 87.7)	

*AUC* area under the curve, *IQR* interquartile range.

**Figure 5:**
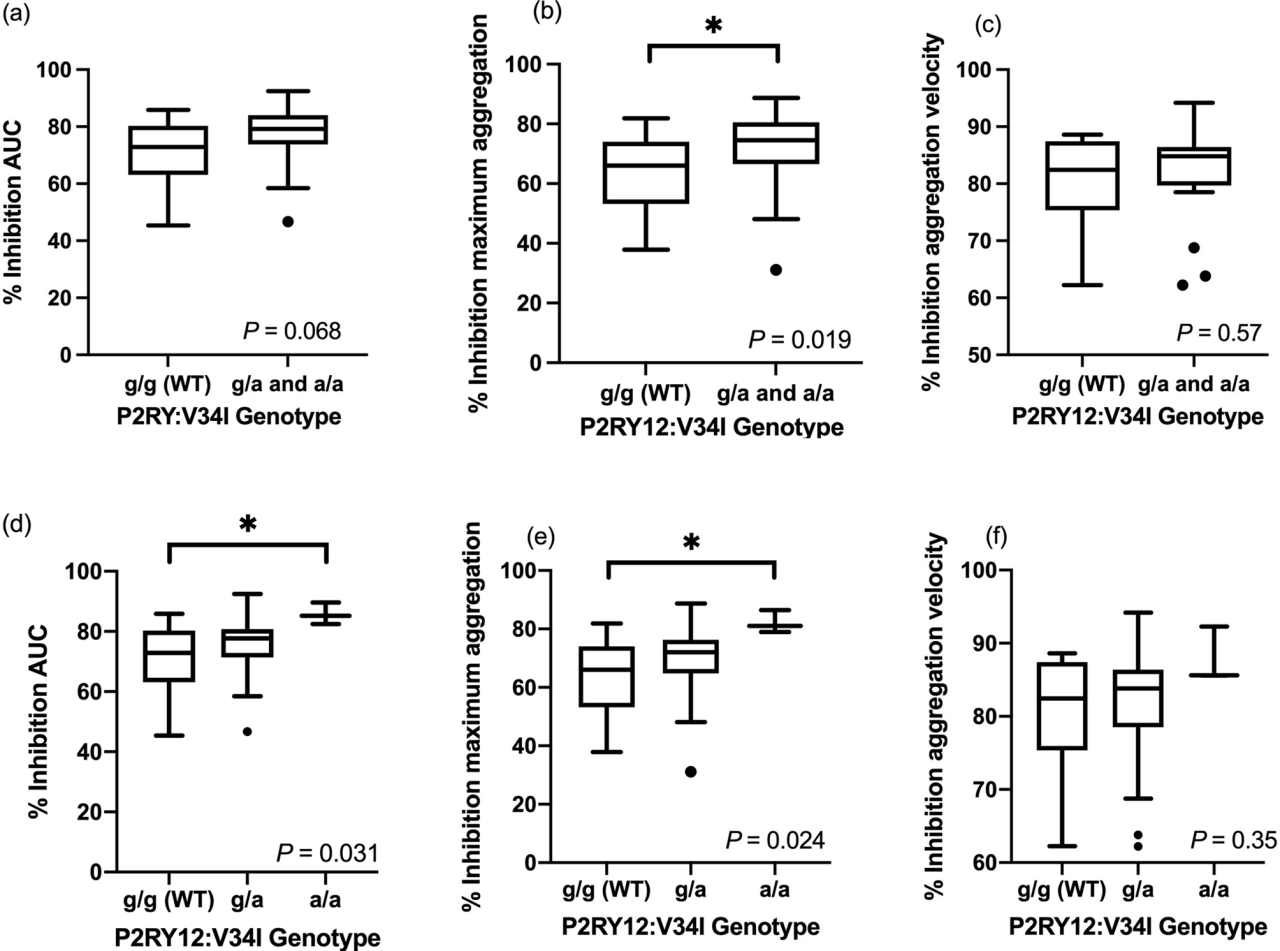
The percent inhibition of (**a**) area under the curve (AUC), (**b**) maximum aggregation (AU), and (**c**) aggregation velocity, measured by whole blood aggregometry, in cats with P2RY12:V34I wildtype (WT) (g/g) and variant genotype (both g/a and a/a genotypes). The percent inhibitions of (**d**) AUC, (e) AU, and (**f**) aggregation velocity were further divided into wildtype (g/g), heterozygous (g/a), and homozygous (a/a) genotypes. The horizontal line represents the median, the box the 25th and 75th percentile, the whiskers the 1.5 times interquartile range, and the points outside the whiskers are outliers.

The percent inhibition of maximum aggregation was significantly lower in cats with the P2RY12:V34I variants (combined homozygous and heterozygous) than cats with P2RY12:V34I wildtype (*P* = 0.019), but this significant difference was not found in AUC (*P* = 0.068) and aggregation velocity (*P* = 0.57) (Fig. 5a–c). When the percent inhibition of AUC was compared among P2RY12:V34I wildtype and heterozygous and homozygous genotypes, it remained significantly different among the three groups (*P* = 0.034). The percent inhibition between the wildtype and homozygous also remained significantly different (*P* = 0.031), whereas no significant difference was noted between the percent inhibition of wildtype and heterozygous genotypes (*P* = 0.96) as well as between heterozygous and homozygous genotypes (*P* = 0.13) (Table 2, Fig. 5d). The same comparisons were made for percent inhibition on maximum aggregation and aggregation velocity among P2RY12:V34I genotypes. When the percent inhibition of maximum aggregation was compared among P2RY12:V34I wildtype and heterozygous and homozygous genotypes, it remained significantly different (*P* = 0.019), and the percent inhibition between the wildtype and homozygous remained significantly different (*P* = 0.024). However, no significant difference was noted between the percent inhibition of wildtype and heterozygous genotypes (*P* = 0.36) as well as between heterozygous and homozygous genotypes (*P* = 0.21) (Table 2, Fig. 5e). When the percent inhibition of aggregation velocity was compared among P2RY12:V34I wildtype and heterozygous and homozygous genotypes, the percent inhibition among the three groups was not significantly different (*P* = 0.35) (Table 2, Fig. 5f).

The CYP2C41:H231R variants were not significantly associated with diminished percent inhibition determined based on platelet aggregometry (Table 2). False discovery rate (FDR) analysis was performed to adjust the multiple comparisons with three genetic polymorphisms. The percent inhibition of AUC (*P* = 0.035) and maximum aggregation (*P* = 0.025) remained significantly lower in cats with P2RY1:A236G variants than the wildtype. On multivariable regression analysis (comparison between wildtype and variants), only P2RY1:A236G remained significantly associated with AUC (*P* = 0.012), maximum aggregation (*P* = 0.015), and aggregation velocity (*P* = 0.032).

**Figure 6:**
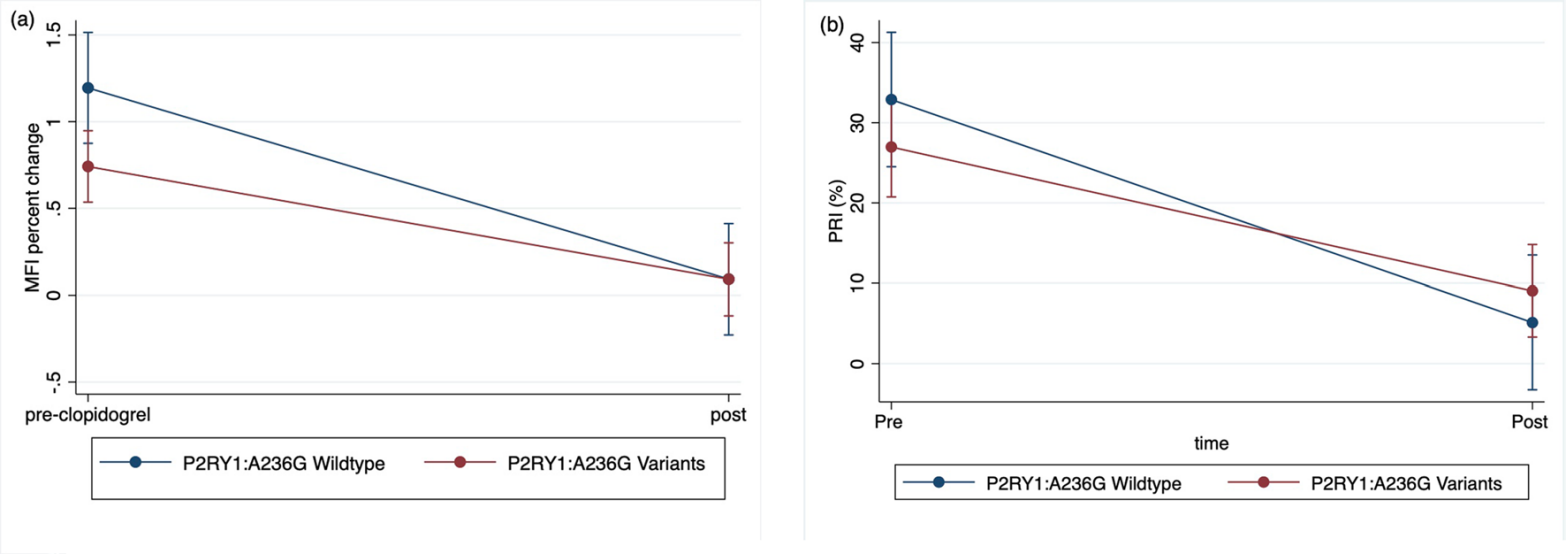
The means and 95% confidence intervals of (**a**) P-selectin mean fluorescence intensity (MFI) percent change and (**b**) platelet reactivity index (PRI) derived from phosphorylated vasodilator-stimulated phosphoprotein (P-VASP) expression before and after 10–14 day course of clopidogrel treatment are noted with a blue solid line for cats with P2RY1:A236G wildtype (c/c) and a red solid lie for the P2RY1:A236G variants (c/g and g/g).

Repeated measure analysis with the linear mixed model was conducted to assess the fixed effect of P2RY1:A236G and P2RY12:V34I variants, time, and the mixed effects of these genetic variant status and time on platelet activation before and after clopidogrel therapy assessed by P-selectin and P-VASP expression. Statistically significant effect with regards to P2RY1:A236G status on P-selectin expression (Fig. 6a), but not on the PRI derived from P-VASP expression (Fig. 6b), was detected. The same analysis was performed for P2RY12:V34I (Supplement Fig. 1) and *CYP2C41* variants but no significant effects with regards to these SNPs on P-selectin and P-VASP expressions were identified.

### Genotypes did not alter clopidogrel metabolite concentrations

Association analysis between the genetic polymorphisms and clopidogrel, clopidogrel metabolites, and CAM-D metabolic ratio were performed. There were no significant differences in clopidogrel, clopidogrel metabolites (carboxylic acid, and CAM-D), and CAM-D metabolic ratio between the wildtype and heterozygous and homozygous variants of CYP2C41:H231R (Supplement Fig. 2). P2RY12:V34I and CYP2C41:H231R missense SNPs were not significantly associated with the concentrations of clopidogrel, clopidogrel metabolites, and CAM-D metabolic ratio.

## Discussion

In this study, the standard dose and frequency of clopidogrel therapy for cats with HCM significantly inhibited platelet function assessed by platelet aggregometry and flow cytometry. The concentrations of clopidogrel and clopidogrel inactive and active metabolites were also successfully measured in all 49 cats. The frequencies of missense SNPs in *P2RY1, P2RY12*, and *CYP2C41* genes in cats with HCM were successfully determined. The phenotypic and genotypic comparisons were then performed for each platelet function parameter and drug concentration and the missense SNPs in these genes. Among these SNPs, P2RY1:A236G and P2RY12:V34I variants were significantly associated with diminished platelet inhibition after clopidogrel administration based on the whole blood impedance aggregometry analysis. The significant association with P2RY1:A236G remained statistically significant even after adjustment for multiple comparisons by FDR analysis. On multivariable analysis, P2RY1:A236G variants also remained significantly predictive of platelet inhibition after clopidogrel administration in all aggregometry parameters.

Activation of the P2Y_1_ receptor on the platelet surface membrane by ADP leads to activation of phosphoinositide-3 kinase and subsequent intracellular calcium mobilization causing shape change and inside-out signaling to activate integrin α_IIb_β_3._ of the other ADP receptor, P2Y_12_, leads to inhibition of adenylyl cyclase hence modulating the production of cyclic adenosine monophosphate (cAMP) resulting in platelet inhibition. Both receptors play important roles in propagating the platelet plug formation by facilitating the binding of fibrinogen to integrin α_IIb_β_3_. Clopidogrel, once metabolized to its active metabolite, selectively inhibits the P2Y_12_ receptor, but not the P2Y_1_ receptor. Therefore, a gain-of-function mutation of the *P2RY*_*1*_ gene could reduce the efficacy of a P2Y_12_ ADP receptor antagonist, leading to clopidogrel resistance. In one human study, a *P2RY1* SNP was associated with a significantly greater reactivity to ADP with due to variants in the *P2RY1* gene^29^. In another human study, however, no difference in the frequency of SNPs in the *P2RY1* gene were reported between clopidogrel responders and non-responders^30^. The different results in terms of the impact of P2RY1 SNPs between these two human studies and our study could be explained by differences in species, underlying diseases, test modalities and study designs. Nevertheless, our study showed that the P2RY1:A236G variant could explain why some cats with HCM do not respond well to clopidogrel.

Whole blood impedance platelet aggregometry, Multiplate, measures the increase in electrical impedance over time as platelets aggregate on a pair of electrodes in response to platelet agonists. This increase in electrical impedance is then measured as maximum aggregation over time. Whole blood impedance platelet aggregometry has been shown to correlate well with light transmission aggregometry which is considered the gold standard for monitoring antiplatelet drugs in humans^31–33^. In addition, some human studies showed that clopidogrel hypo-responsiveness as determined by whole blood aggregometry was an independent predictor for developing thromboembolic events after percutaneous coronary intervention procedures^34,35^. In cats, whole blood impedance aggregometry was previously validated as a monitoring tool for several different antiplatelet and anticoagulant drugs including clopidogrel^15,36,37^. Variants in the *P2RY1* gene may lead to either upregulation or gain-of-function of P2Y_1_ receptor, which is coupled to the G protein, G_q_. Once activated, it provides a robust stimulation of phosphatidylinositol hydrolysis by phospholipase C resulting in a rise in cytosolic calcium, which mediates several crucial events including activation of integrin α_IIb_β_3,_ switching it from a low to high affinity state. Increased response to a physiologic concentration of ADP may further increase receptor affinity to fibrinogen causing faster and more extensive platelet aggregate formation. Decreased inhibition in aggregation velocities and total aggregation in cats with P2RY1:A236G genotypes likely explain their augmented response to ADP in whole blood aggregometry. The phenotype of this variant, however, requires further characterization.

It is thus reasonable to utilize the Multiplate aggregometry to determine clopidogrel responsiveness in this study population of cats with HCM, and the significant association between the P2RY1:A236G genotypes is likely a true finding.

Significant association between platelet inhibition by clopidogrel in cats with the P2RY1:A236G variant was not observed when P2Y_12_ inhibition was assessed by changes in intraplatelet P-VASP. Measurement of P-VASP is used as a specific marker of P2Y_12_ antagonism in people and this test was recently validated in cats^38–40^. In our study, ADP activation of P2Y_12_ significantly decreased intraplatelet P-VASP expression with the PGE1 stimulation, and clopidogrel treatment resulted in sustained P-VASP expression indicating specific inhibition of P2Y_12_ receptor. However, there was a considerable variability of PRI in this cohort of HCM affected cats, and this finding might support an interindividual variability of clopidogrel responsiveness in cats with HCM when P-VASP expression was assessed before and after clopidogrel therapy. However, this variability was not significantly associated with any nonsynonymous SNP in the *P2RY12* gene we investigated. This finding supports the idea that the tested missense SNPs in *P2RY12* gene are less likely to have a primary responsibility for interindividual variability of clopidogrel responsiveness in cats with HCM. In addition, nonsignificant association between *P2RY1* genotypes and P-VASP expression were found in our study. This could imply that P-VASP expression analysis might not be the best modality to identify feline clopidogrel non-responders. This result might also indicate that other genotypic or phenotypic variability influences clopidogrel responsiveness causing a considerably large interindividual variability of PRI after clopidogrel therapy.

P-selectin expression could be utilized as a marker of platelet activation induced by ADP and subsequent alpha-granule secretion. In previous studies, this modality was validated to assess platelet function and clopidogrel responsiveness in cats with HCM^14,41^. Significant attenuation of P-selectin expression by clopidogrel observed in our study indicates that this could be used as another monitoring tool for the efficacy of clopidogrel therapy. However, similar to P-VASP, no significant association was noted between the P-selectin expression and any genotypes including *P2RY1* and *P2RY12* variants. This result could be explained by the fact that alpha-granule secretion in platelets is induced by not only ADP but also various agonists, and genotypic impact on clopidogrel responsiveness could be masked by up-regulation of other platelet activation pathways.

In people, genetic variants in relation with decreased metabolism of clopidogrel to an active metabolite are the most frequently attributed mutations leading to clopidogrel resistance^16–19,21–23^. In our study, the concentration of CAM-D was measured, and CAM-D metabolic ratio was calculated based on the concentrations of clopidogrel and clopidogrel metabolites. In humans, several studies supported that decreased CAM-D concentration was associated with clopidogrel resistance due to decreased metabolism of clopidogrel to the active metabolite^22,42–44^. In cats, one recent study showed that the major contributing factor for interindividual variability of clopidogrel response after one dose of clopidogrel was at least partially explained by difference in clopidogrel metabolism^28^. In addition, the same study reported that one of the missense SNPs in the *CYP2C41* gene, CYP2C41:H231R, was significantly associated with increased CAM-D concentrations and metabolic ratio in cats with the homozygous variant. This finding indicated that the CYP2C41:H231R homozygous variant increases the metabolism of clopidogrel and could lead to an increased risk of major bleeding events. However, no significant correlation was noted between platelet inhibitory effect of clopidogrel and CAM-D as well as CAM-D metabolic ratio in our study. In addition, CAM-D and CAM-D metabolic ratio did not show significant association with any of genetic variants including CYP2C41:H231R. The discrepancy of these findings in the previous and our studies could be explained by the duration of clopidogrel therapy provided to cats. In the previous study, clopidogrel metabolite concentrations and CAM-D metabolic ratio were obtained after one dose of clopidogrel, whereas these were obtained after a 10–14 day course of clopidogrel therapy in our study. This duration of clopidogrel therapy could be long enough to saturate the cytochrome P450 enzyme thus minimizing the effects of the CYP2C41:H231R variant on the CAM-D concentrations. It should be also noted that only four out of 49 cats enrolled in our study was considered as homozygous variant with CYP2C41:H231R genotypes. This low allelic frequency is consistent with the findings in the previous study where only three out of 19 cats were reported to be a CYP2C41:H231R homozygous. The low sample size for this specific variant could lead to either false positive results in the former study or false negative results in our study when investigating associations with the CYP2C41:H231R variant and CAM-D concentration.

There are a few limitations of our study. First, although our study was a prospective clinical trial, there are still relatively large numbers of confounders including breeds, age, weight, and severity of HCM. These confounders might indicate a requirement of a larger sample size than we initially calculated based on a priori power calculations. The significant association between clopidogrel responsiveness and P2RY1:A236G genotypes is likely a true positive due to a persistent significance despite FDR adjustment. Also, the multivariable regression analysis reported that the P2RY1:A236G genotype significantly predict clopidogrel responsiveness in these cats with HCM. Secondly, drug administration was performed by the owners and thus we cannot confirm beyond the owner’s verbal assurance that all cats definitively received clopidogrel as directed for 10–14 days. Failure to follow the protocol could influence the effects of clopidogrel on platelet function and the concentration of clopidogrel and clopidogrel metabolites after the therapy. Since we were successful at identifying and excluding cats with difficulty or unable to administer clopidogrel at home were successfully identified and these cats were excluded from the analyses, owner compliance is assumed to be a minimal problem in our study. Third, only SNPs in the exon region of the genes of interest were investigated in our study, and it is possible that other genes and SNPs in non-exonic regions could also influence clopidogrel responsiveness. Therefore, further investigation is warranted to determine if there are any other variants that modify clopidogrel responsiveness in cats. Finally, it is unclear if P2RY1:A236G variant leads to clinically increased frequency of developing cardiogenic ATE in spite of clopidogrel, and a longitudinal observational study should be conducted to determine if on-treatment thromboembolic complications occur more frequently in HCM cats with P2RY1:A236G variant. Nevertheless, it appears that the *P2RY1* genetic mutation is associated with decreased efficacy of clopidogrel.

This study demonstrated significant inhibition of ADP-induced platelet aggregation, P-selectin expression, and P-VASP expression in cats with HCM after 10–14 days of clopidogrel therapy. More importantly, platelet inhibition by clopidogrel in cats with the P2RY1:A236G variant was significantly diminished compared to cats with the P2RY1:A236G wildtype genotype. This result supports our hypothesis that a SNP within the ADP receptor (P2Y_1_) gene contributes to clopidogrel resistance in cats with HCM. Although further prospective study is necessary, the findings of our study may suggest that genotype might become one of the factors that should be considered when selecting antithrombotic therapy in cats with HCM. This data underscores a role for personalized medicine and genetic testing in the selection of therapies for cats at risk of thromboembolic disease.

## Methods and materials

### Animals and study design

This study was approved by the Animal Care and Use Committee of the University of California-Davis and performed in accordance with relevant guidelines and regulations. This study was carried out in compliance with the ARRIVE guidelines. Informed owner consent was obtained for all cats prior to enrollment. All cats were enrolled between September 2018 and September 2019. Cats diagnosed with HCM were enrolled following echocardiographic verification of a diagnosis of HCM. All screened cats underwent a general health assessment, which consisted of cardiovascular physical examination, a complete echocardiographic examination, Doppler sphygmomanometry for systolic blood pressure measurement, complete blood count, serum biochemistry and total thyroxine (T4) measurement (Vetscan VS2 Chemistry Analyzer, Abaxis, Abbott Group, Union City, CA). Cats with any cardiac disease other than HCM were excluded from this study. Other exclusion criteria included any clinically apparent systemic diseases including abnormal findings on complete blood count and serum biochemistry, systemic hypertension (systolic blood pressure > 170 mmHg), hyperthyroidism, or any clinically significant arrhythmia. In addition, cats taking any medications known to affect platelet function and coagulation as well as cytochrome P-450 enzyme activity were excluded. Cats that were noncompliant or difficult to safely handle and restrain without any oral or parenteral sedation were also excluded from the study. Cats were allowed to receive furosemide, angiotensin-converting enzyme inhibitors, and atenolol, but the doses and frequencies of these medications were unchanged throughout the study period. The enrolled cats were then treated with clopidogrel 18.75 mg orally every 24 h for 10–14 days. The ones that developed any clinical signs of congestive heart failure, such as respiratory signs, during the study period and/or exhibited reported adverse effects of clopidogrel, such as vomiting, diarrhea, inappetence, and bleeding, were excluded. Cats that were difficult to be medicated at home were removed from the study and excluded from the study analysis. The enrolled cats completing the clopidogrel treatment period were reevaluated after 10–14 days of clopidogrel therapy.

### Echocardiographic examination

Complete echocardiographic examination was performed by an American College of Veterinary Internal Medicine board-certified cardiologist (JS, MO), or a cardiology resident or a cardiology research fellow directly supervised by the cardiologist. A 4- to 12-mHz sector-array transducer (S12–4) was used for all echocardiographic examinations. All echocardiographic examinations were successfully performed with gentle restrain without sedation. All measurements were performed using an offline analysis software (Syngo Dynamics, Siemens, Erlangen, Germany). HCM was diagnosed if cats had interventricular and/or left ventricular posterior wall thickness exceeding 6 mm^45,46^. Cats with changes consistent with HCM were kept in lateral recumbency after echocardiography, and their systolic blood pressure was obtained using a Doppler blood pressure measurement device and sphygmomanometer to rule out possible systemic hypertension as an inciting cause of left ventricular hypertrophy. Systemic hypertension was suspected if systolic blood pressure persistently exceeded 160 mmHg and the cats with persistent systemic hypertension were excluded from this study. After an assessment of echocardiographic images, ECG, systolic blood pressure complete blood count, biochemical profile and total T4 level, cats with HCM and without any exclusion criteria were enrolled in the study.

### Blood sampling

Using a 21-gauge butterfly catheter, six milliliters of blood were obtained from the medial saphenous vein. The sample was immediately aliquoted to 3.2% trisodium citrate tubes (BD Vacutainer CTAD tubes), sodium heparin (BD Microtainer), and tubes containing recombinant hirudin (S-Monovette, Sarstedt AG & Co. Numbrecht, Germany). Complete blood count, serum biochemistry and T4 concentration were measured using automated analyzers (HM5/VetScan2 Abaxis, Union City, CA). Cats without any apparent abnormalities in these blood tests were enrolled in the study. Six milliliters of blood samples were again collected approximately 2 h after the last study dose of clopidogrel given 10–14 days after enrollment. An additional one ml of whole blood was obtained and placed in a 1.5 mL conical tube containing mass spectrometry compatible reagents for measuring clopidogrel and clopidogrel metabolite concentrations as previously described^28^.

### Whole blood platelet aggregometry

Blood samples in the hirudin-anticoagulant tube was kept in a 37 °C bead bath for 30 min immediately after blood collection. The sample was then used for assessing platelet aggregation by whole blood multiple electrode impedance aggregometry (Multiplate, Roche Diagnostics GmbH, Mannheim, Germany). Briefly, 300 µL of pre-warmed 0.9% sodium chloride solution was added into the test cell preheated to 37 °C. The blood sample (300 µL) was then added to the test cell. The diluted blood sample was incubated at 37 °C for 3 min under physiologic shear stress generated by a Teflon-coated magnetic stir bar at 800 rpm. ADP (6 µM) was then added and impedance was recorded for 6 min. The results are based on platelet aggregation, which occurred on the silver-coated electrodes within each test cell resulting in the increase in electrical impedance. Results were reported as the area under the curve values (AUS; AU * min), maximum aggregation (AU), and aggregation velocity (AU/min). Percent inhibition was calculated based on ADP-induced aggregation (ADP-ag) before and after clopidogrel therapy using the following formula^10,38^:

$${\text{Percent}}\;{\text{ inhibition }}\left( \% \right) \, = \left( {\frac{{ADP - ag\left( {pre - clopidogrel} \right) - ADP - ag \left( {post - clopidogrel} \right)}}{{ADP - ag \left( {pre - clopidogrel} \right)}}} \right) \times 100$$

### Platelet P-selectin expression detected by flow cytometry

Blood samples were transferred from 3.2% trisodium citrate tubes to polypropylene tubes before placing them in a 37 °C bead bath for 30 min. The samples were then centrifuged in 25 to 27 °C at 200 × g for 5 min to generate platelet-rich plasma (PRP). PRP was used to analyze platelet function within 2 h after blood sample collection. The concentration of platelet in PRP was standardized to 1 × 10^7^ cells/mL with a final volume of 100 µL by diluting PRP with Tyrodes-HEPES (5 mM dextrose, pH 7.2, no divalent cations). Platelets in PRP were either unstimulated (resting) or stimulated by 20 µM ADP (MilliporeSigma, Burlington, MA), and then incubated for 15 min at 37 °C. P-selectin on the surface of activated platelets was labeled with monoclonal rat anti-mouse antibody that was conjugated with fluorescein isothiocyanate (1:200, clone RB40.34, BD Pharmingen, San Jose, CA) by incubation for 45 min at 37ºC. The labeled platelets were detected by forward and side scatter properties calibrated by 0.9 µm and 3 µm beads, as well as integrin beta-3 (CD61) identified using allophycocyanin-conjugated polyclonal mouse anti-human antibody (1:1000, clone VI-PL2, Invitrogen, Carlsbed, CA). All antibodies were previously validated to cross-react with feline platelets. Samples were fixed with 1% paraformaldehyde and analyzed using a 5-color follow cytometer (Beckman-Coulter FC500, Miami, FL). Monoclonal mouse immunoglobulin G1 kappa and anti-mouse compensation beads that were conjugated to matched experimental fluorochromes were used as compensation controls. Gating boundaries were created fluorescence-minus-one controls to determine CD62P and CD61 positive events within the platelet gate.

### Intraplatelet P-VASP expression detected by flow cytometry

Intraplatelet P-VASP was measured in unstimulated (resting) platelets, or platelets treated with 20 μM ADP (MilliporeSigma, Burlington, MA), 5 µM PGE_1_ (MilliporeSigma, Burlington, MA), or a combination of 20 µM ADP and 5 µM PGE_1_ for 15 min at 37 °C as previously described^38^. In brief, the treated platelets were first fixed with 1% methanol-free paraformaldehyde for 15 min at room temperature, permeabilized with 0.25% detergent (NP-40 Surface-AMPs Detergent Solution, Thermo Fisher) then centrifuged at 5000 × *g* for 1 min at room temperature. After supernatant was discarded, 100µL of Tyrodes HEPES was added to the tube to resuspend the pellets containing the permeabilized platelets. P-VASP was labelled by mouse polyclonal antibody conjugated to fluorescein isothiocyanate (5 ug/mL, ALX-804-240F-C100, Enzo Life Sciences, Farmingdale, NY) and incubated at room temperature with light protection for 90 min. P-VASP expression was detected by flow cytometry as previously described^38^. Flow cytometry data was analyzed using a commercially available software (FlowJo, Tree Star Inc, Ashland, OR). The magnitude of platelet inhibition was expressed as platelet reactivity index (PRI), calculated using the equation below^47^:

$${\text{PRI }} = \left( {\frac{{MFI\left( {PGE1} \right) - MFI\left( {PGE1 + ADP} \right)}}{{MFI\left( {PGE1} \right)}}} \right) \times 100$$

### Clopidogrel active metabolite measurement

Immediately after blood draw from the cats, one milliliter of whole blood was placed in a two-milliliter conical tube containing mass spectrometry compatible reagents, which consist of 20 µL of a 500 mM EDTA solution and 20 µL of 500 mM racemic (e)-2-bromo-3′-methocyacetophenone (BMAP) (Sigma-Aldrich, St. Louis, MO, USA). The 500 mM BMAP was made by mixing 1 mL of acetonitrile (Fisher Scientific, Pittsburgh, PA, USA) and 115 mg of BMAP. After gently inverting the tube multiple times to mix the blood sample and the reagents, the tube was kept in the ice until it was processed within thirty minutes after sample collection. The sample was then centrifuged at 15,000 g for 1 min, and the supernatant was transferred to a new 1.5-mlliliter conical tube and stored in − 80 °C until its used. Plasma concentrations of clopidogrel and clopidogrel metabolites were measured using an Agilent 1100 series HPLC coupled to an Applied Biosystems API 4000 LC/MS/MS tandem mass spectrometer system. A Synergi Phenomenex Polar-RP 2.0 × 150 mm column was used for separation at a column temperature of 30 °C. After measuring the clopidogrel and clopidogrel metabolite concentrations, CAM-D metabolic ratio was calculated using the equation below^28^.

$${\text{CAM}} - {\text{D}}\;{\text{metabolic}}\;{\text{ratio}} = \left( {\frac{{{\text{CAM}} - {\text{D molarity}}}}{{{\text{Clopidogrel }}\;{\text{molarity}} + {\text{clopidogrel }}\;{\text{carboxylic }}\;{\text{acid }}\;{\text{molarity}}}}} \right) \times 100$$

### DNA sequencing

According to the manufacture’s protocol, genomic DNA was extracted from hirudin or 3.8% trisodium citrate blood samples using a commercially available kit (Puregene, Gentra Systems, Minneapolis, MN). The PCR primers were selected from previous studies^25,28^. SNPs were then assayed at University of California-Davis, the Veterinary Genetics Laboratory, using a Sequenom MassARRAY Compact 96 with iPLEX Gold technology (Sequenom, San Diego, CA, USA). Frequencies of SNPs in *P2RY1, P2RY12*, and *CYP2C41* genes were calculated based on the alignment results. Amplicons were aligned to the cat reference sequence, ICGSC Felis-catus_8.0/felCat8, using DNASTART LASERGENE (DNASTAR, Inc.).

### Statistical analysis

A priori power calculations were performed. We used mutation frequency data available from a cohort of 83 unrelated cats of multiple breeds to confirm that the investigated SNPs were predicted to have a frequency of at least 10% permitting appropriate power of this. Given a two-tailed design with an alpha error = 0.05, and an effect size beyond biologic variability at 20% we expected to identify significant changes with an 80% power. This calculation identified a need for 49 cats. Each cat served as its own pre-clopidogrel control in order to determine response to therapy. Genotype groups for each variant were treated in a pooled (heterozygous and homozygous variant as a single group versus wildtype) and individual (homozygous vs. heterozygous vs. wildtype) fashion. Normality was tested by D’Agostino normality test. The variables were reported with mean and standard deviation, or median and interquartile range depending on their distributions. Comparisons of two groups with continuous variables were performed by student’s t-test or Mann–Whitney test. Repeated measures were compared by paired t-test or Wilcoxon matched-pairs signed-ranks test between two groups. The repeated measures were compared among more than three groups by repeated measure of ANOVA or Friedman test followed by post–hoc Tukey or Dunn’s multiple comparison test, respectively. Pearson’s correlation analysis was performed between platelet function variables and clopidogrel metabolite concentrations. All genetic variants and the whole blood aggregometry results were included for multivariable analysis. A stepwise selection technique was employed by sequentially adding the variables if *P*-value is less than 0.1. Missense SNPs were tested for association with altered platelet aggregation inhibition by clopidogrel based on the results from whole blood aggregometry analyzer, platelet activation assessed by P-VASP and P-selectin expressions before and after clopidogrel therapy by performing repeated measures with a linear mixed model. To avoid Type-I error and decreasing the false discovery rate (FDR) due to multiple comparisons, the Benjamini–Hochberg procedure was performed by including all four SNPs in the calculation. Chi-square analysis for goodness of fit was performed to verify deviation from Hardy–Weinberg equilibrium.

## Supplementary Information

Supplementary Information.

## Data Availability

The authors declare that all data supporting the findings of this study are available within the article or from the corresponding author upon request.
