# Supplementary material for: A genetic polymorphism in P2RY1 impacts response to clopidogrel in cats with hypertrophic cardiomyopathy
Source: Sci Rep. 2021 Jun 15;11:12522. doi: 10.1038/s41598-021-91372-3 (PMC8206363; doi:10.1038/s41598-021-91372-3)

**A genetic polymorphism in *P2RY_1_* impacts response to clopidogrel in cats with hypertrophic cardiomyopathy**

Yu Ueda^1^, Ronal H.L. Li^2^, Nghi Nguyen^2^, Eric S. Ontiveros^3^, Samantha L. Fousse^3^, Maureen S Oldach^2^, Karen M. Vernau^2^, Michael H. Court^4^, Joshua A. Stern^3^

1. *North Carolina State University, College of Veterinary Medicine, Department of Clinical Sciences, Raleigh, NC, USA*
2. *University of California – Davis, School of Veterinary Medicine, Department of Veterinary Surgical and Radiological Sciences, Davis, CA, USA*
3. *University of California – Davis, School of Veterinary Medicine, Department of Medicine and Epidemiology, Davis, CA, USA*
4. *Washington State University, College of Veterinary Medicine, Department of Veterinary Clinical Sciences, Pullman, WA, USA*

**Supplement Table 1.** Correlation analyses between platelet function tests and clopidogrel and clopidogrel metabolite concentrations measured after the 10-14 days of clopidogrel therapy in cats with HCM are listed as correlation coefficient (r) and the corresponding *p-*value. The values with a significant correlation (*p* < 0.05) are shown with asterisks.

|  |  | Clopidogrel (ng/mL) | Clopidogrel acid (ng/mL) | CAM-D (ng/mL) | CAM-D metabolic ratio |
| --- | --- | --- | --- | --- | --- |
| Multiplate AUC % inhibition | *r* | -0.0039 | 0.15 | 0.24 | 0.088 |
|  | *p*-value | 0.98 | 0.34 | 0.12 | 0.58 |
| Multiplate AUC post-clopidogrel | *r* | -0.016 | -0.2 | -0.2 | 0.063 |
|  | *p*-value | 0.92 | 0.19 | 0.18 | 0.69 |
| Multiplate Aggregation % inhibition | *r* | 0.0067 | 0.15 | 0.22 | 0.1 |
|  | *p*-value | 0.97 | 0.35 | 0.15 | 0.51 |
| Multiplate Aggregation post-clopidogrel | *r* | 0.017 | -0.15 | -0.16 | 0.024 |
|  | *p*-value | 0.91 | 0.32 | 0.32 | 0.88 |
| Multiplate Velocity % inhibition | *r* | -0.074 | 0.055 | 0.14 | 0.1 |
|  | *p*-value | 0.64 | 0.73 | 0.37 | 0.51 |
| Multiplate Velocity post-clopidogrel | *r* | 0.19 | -0.26 | -0.2 | 0.63* |
|  | *p*-value | 0.2 | 0.07 | 0.17 | 0.0001 |
| PVASP PRI % inhibition | *r* | 0.14 | 0.24 | 0.033 | -0.13 |
|  | *p*-value | 0.39 | 0.14 | 0.84 | 0.41 |
| PVASP MFI post-clopidogrel | *r* | -0.18 | -0.35* | -0.078 | 0.27 |
|  | *p*-value | 0.23 | 0.0015 | 0.6 | 0.061 |
| P-selectin % change of % change | *r* | -0.1 | 0.075 | 0.0063 | 0.31* |
|  | *p*-value | 0.53 | 0.65 | 0.97 | 0.046 |
| P-selectin % change post-clopidogrel | *r* | 0.15 | 0.12 | -0.13 | 0.31* |
|  | *p*-value | 0.36 | 0.45 | 0.43 | 0.046 |

**Supplement Figure 1.** The means and 95% confidence intervals of (a) P-selectin percent change and (b) PRI derived from phosphorylated vasodilator-stimulated phosphoprotein (P-VASP) expression before and after 10-14 day course of clopidogrel treatment are noted with a blue solid line for cats with P2RY12:V34I wildtype (g/g) and a red solid lie for the P2RY12: V34I variants (g/a and a/a).


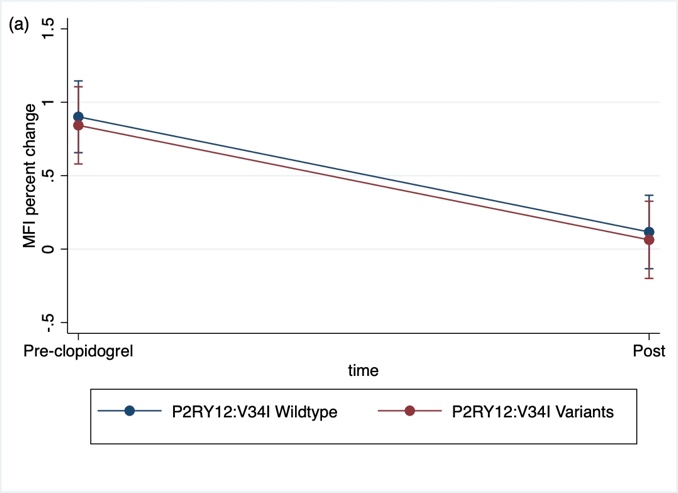

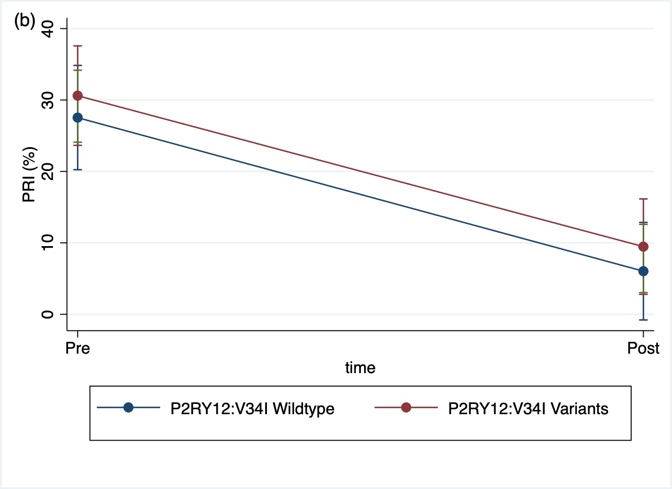


**Supplement Figure 2.** Comparison of (a) plasma derivatized clopidogrel active metabolite (CAM-D) concentrations and (b) calculated CAM-D metabolic ratio between different variants for the CYP2C41:H231R. The horizontal line represents the median, the box the 25^th^ and 75^th^ percentile, the whiskers the 1.5 times interquartile range, and the points outside the whiskers are outliers.


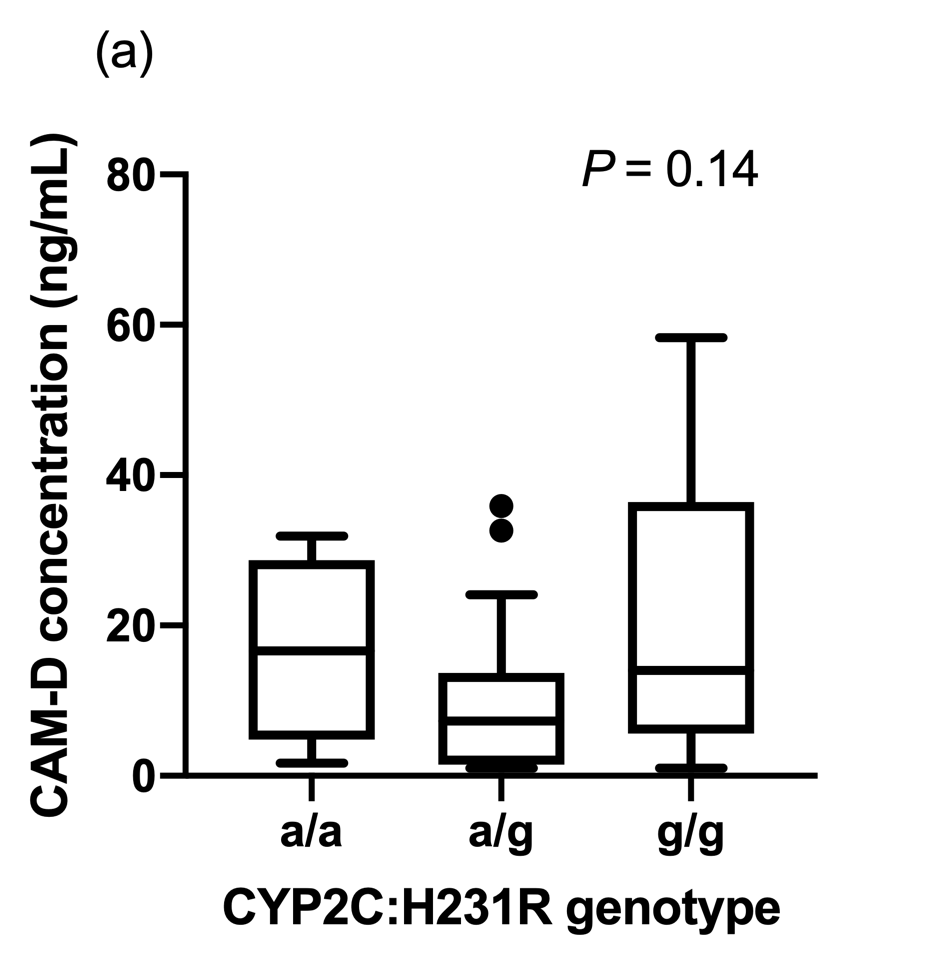

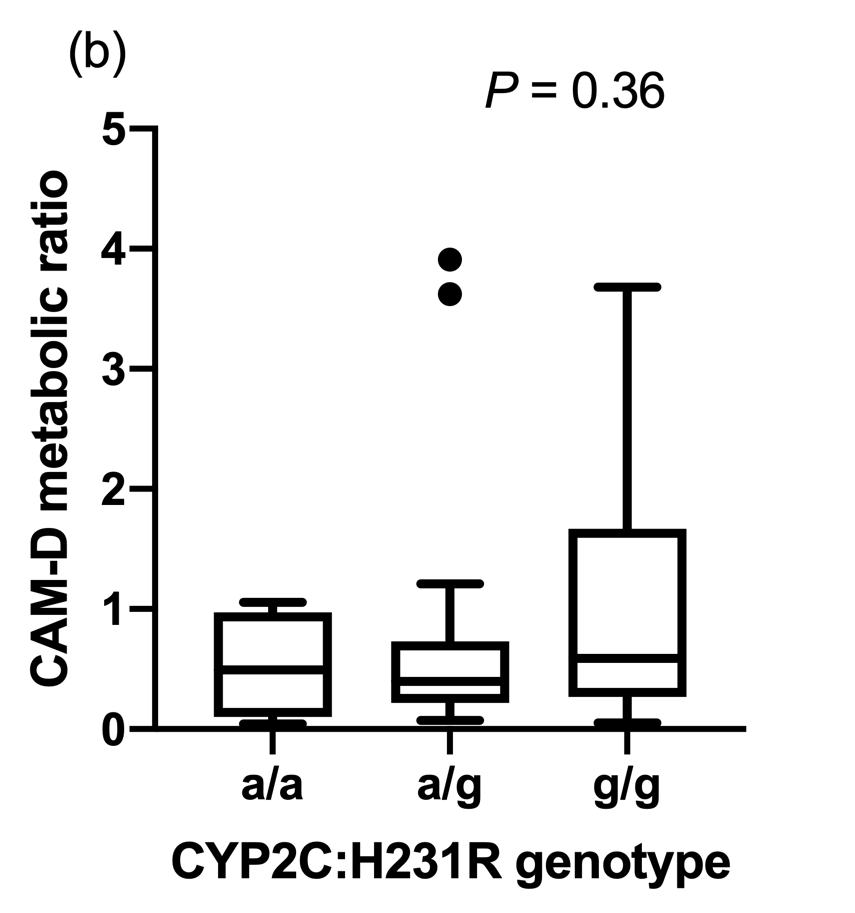

Supplement: Supplementary file 1 — Supplementary Information. [file 41598_2021_91372_MOESM1_ESM.docx]
